# Severe Malaria and Mechanical Ventilation in Pregnancy: The Crucial Role of Fetal Doppler in Monitoring Fetal Well-Being

**DOI:** 10.7759/cureus.98109

**Published:** 2025-11-29

**Authors:** Sandra Olaya, Sergio David Angulo, Jezid Miranda, Manuel Garay

**Affiliations:** 1 Gynecology, Fundación Universitaria Autónoma de las América, Pereira, COL; 2 Gynecology, Clinica Comfamiliar, Pereira, COL; 3 Gynecology, Clínica Comfamiliar, Pereira, COL; 4 Research and Teaching Unit, Salud Comfamiliar, Pereira, COL; 5 Obstetrics and Gynaecology, Universidad de Cartagena, Cartagena, COL; 6 Gynecology, Centro Hospitalario Serena del Mar, Cartagena, Colombia y Fundación Santa Fe de Bogotá, Cartagena, COL; 7 Critical Care, Hospital Santa Clara, Bogota, COL

**Keywords:** doppler, fetal wellbeing, malaria, mechanical ventilation, pregnancy

## Abstract

A 33-year-old multiparous woman with no relevant medical history, at 33 weeks of gestation, presented to the emergency department of a tertiary care hospital with a five-day history of fatigue, arthralgia, chills, and abdominal pain. The patient rapidly developed respiratory failure and was intubated and placed on mechanical ventilation. Continuous fetal monitoring, including Doppler velocimetry, was performed to assess fetal well-being during maternal respiratory support. Fetal Doppler evaluations demonstrated reassuring parameters during mechanical ventilation, with normal umbilical artery and middle cerebral artery flow patterns. No signs of fetal hypoxia or centralization were observed. A review of the literature identified fewer than a dozen case reports or series involving mechanical ventilation in pregnant patients, most commonly associated with severe pneumonia, acute respiratory distress syndrome, or trauma. Few studies addressed fetal monitoring strategies or outcomes related to Doppler indices during maternal ventilation. This case highlights the feasibility and importance of fetal Doppler surveillance in pregnant patients undergoing invasive mechanical ventilation. Despite limited data, current evidence suggests that individualized maternal ventilatory management, coupled with serial fetal assessment, can support favorable perinatal outcomes. Further studies are needed to define optimal respiratory parameters and standardized fetal monitoring protocols in this unique clinical scenario.

## Introduction

Mechanical ventilation is a life-sustaining intervention used in critically ill patients who are unable to maintain adequate spontaneous respiration [1]. Before the COVID-19 pandemic, respiratory failure that required mechanical ventilator support in pregnancy was a relatively uncommon event, occurring at a rate of approximately 1 in 10,000 pregnancies [2]. While well characterized in the general population, its use during pregnancy poses unique challenges due to physiological changes in maternal respiratory and cardiovascular function [3,4]. These adaptations can influence the fetoplacental circulation and the ability to deliver oxygen and nutrients to the developing fetus. There are no standardized guidelines regarding how to provide mechanical ventilation in pregnant patients. We present a case of a pregnant woman at 33 weeks of gestation requiring invasive mechanical ventilation, with a focus on fetal Doppler assessment during respiratory support.

## Case presentation

A 33-year-old multiparous woman with no relevant medical history, at 33 weeks of gestation, presented to the emergency department of a tertiary care hospital with a five-day history of fatigue, arthralgia, chills, and abdominal pain. On initial evaluation, the patient appeared acutely ill, with mucocutaneous pallor and prolonged capillary refill time. Vital signs revealed hemodynamic instability. The patient rapidly developed respiratory failure and was intubated and placed on mechanical ventilation, and laboratory data are shown in Table 1. The peripheral smear confirmed *Plasmodium falciparum* trophozoites, and additional findings revealed multiorgan dysfunction.

**Table 1:** Laboratory data at admission AST: aspartate aminotransferase, ALT: alanine aminotransferase, pCO₂: partial pressure of carbon dioxide, paO₂: partial pressure of arterial oxygen, HCO₃: bicarbonate, BE: base excess, SO₂: oxygen saturation, PAFI: paO₂/FiO₂ ratio.

Variables	Patient	Reference range
Hemoglobin (g/L)	10.1	11.4–14.7
Mean corpuscular volume (Fl)	75	80–100
Hematocrit	30.5%	36.9–54.3%
White blood cells/μL	7840	3500–13,500
Platelet count (cells/μL)	90,000	150,000–450,000
Creatinine (mg/dL)	1.8	1.1
Blood urea nitrogen (mg/dL)	46	6–20
AST (U/L)	150	5–34
ALT (U/L)	190	0–55
Total bilirubin (mg/dL)	2.4	0.1–1
Arterial blood gases		
pH	7.3	7.35–7.45
pCO_2_ (mmHg)	27	35–45
paO_2_ (mmHg)	101	80–100
HCO_3_ (mmol/L)	16	22–29|
BE (mmol/L)	–9.4	–2 to +2
SO_2_	97%	>92%
PAFI	482	>400

The pregnant woman was transferred to the intensive care unit (ICU), requiring antimalarials (artemether-lumefantrine), invasive mechanical ventilation, vasopressor support, and sedation. The patient received invasive ventilatory support in controlled mode under neuromuscular relaxation and during an alveolar recruitment strategy. Mechanical ventilation was delivered in controlled mode, including an alveolar recruitment maneuver with incremental positive end-expiratory pressure (PEEP). The corresponding ventilatory parameters during the maneuver are presented in Table 2.

**Table 2:** Alveolar recruitment parameters PBW: predicted body weight.

PEEP level (cmH2O)	Tidal volume (mL/kg (PBW))	Pressure plateau (cmH2O)	Compliance (mL/cmH2O)	Driving pressure (cmH2O)
6	310	12	22	6
8	310	13	24	5
10	310	15	21	5
12	310	19	21	7
14	310	22	18	12
16	310	21	20	5

Fetal monitoring and Doppler assessment

Given the maternal critical condition, continuous fetal monitoring was performed to assess fetal well-being during invasive ventilation. Surveillance included electronic fetal heart rate monitoring, biophysical profile, and fetoplacental Doppler velocimetry. Initially, the fetal heart rate and biophysical profile were reassuring, but a non-reassuring fetal status (NRFS) was identified due to reduced variability on the fetal heart tracing.

The first fetoplacental Doppler ultrasound was conducted under baseline ventilatory settings, including a PEEP of 8 cmH₂O. This was followed by an alveolar recruitment strategy, increasing PEEP stepwise from 6 to 16 cmH₂O. A second Doppler assessment was performed at PEEP 16 cmH₂O. Throughout the procedure, the mother maintained adequate hemodynamic stability and oxygenation without signs of decompensation. The Doppler parameters before and after recruitment are summarized in Table 3.

**Table 3:** Fetal Doppler and maternal ventilation parameters before and during recruitment maneuver. PEEP: positive end-expiratory pressure; FiO₂: fraction of inspired oxygen; RR: respiratory rate; PI: pulsatility index; CPR: cerebroplacental ratio (MCA PI/UA PI); P5–P95: Between 5th and 95th percentile for gestational age.

Timing	Ventilation parameters	Fetal Doppler	Doppler findings	Interpretation
Before recruitment	PEEP: 8 cmH2O; FiO2: 35%; RR: 18 bpm; tidal volume: 310 mL	Middle cerebral artery	PI: 2.24 (within normal range, P5–P95); normal morphology	Normal cerebral perfusion
		Umbilical artery	PI: 1.02 (within normal range, P5–P95); umbilical vein with normal morphology	Normal placental-fetal circulation
		Cerebroplacental ratio	CPR: 2.19 (normal)	Adequate brain-sparing not required
		Ductus venosus	PI: 0.67 (within normal range, P5–P95); normal waveforms	Normal venous return
During recruitment	PEEP: 16 cmH2O; FiO2: 35%; RR: 18 bpm; tidal volume: 310 mL	Middle cerebral artery	PI: 1.15 (below P5); signs of cerebral vasodilation	Suggestive of fetal adaptation to hypoxia
		Umbilical artery	PI: 1.30 (above P95); increased placental resistance	Evidence of uteroplacental insufficiency
		Cerebroplacental ratio (CPR)	CPR < 1	Abnormal brain-sparing response
		Ductus venosus	PI: 0.75 (within normal range, P5–P95); preserved waveform	Normal cardiac preload and venous return

Pregnancy outcome

After completing a three-day course of antimalarial therapy and achieving clinical stabilization in the ICU, the patient was discharged for routine outpatient follow-up. She subsequently experienced a spontaneous, full-term vaginal delivery without complications. The neonate weighed 3.30 kg, with Apgar scores of 8 at one minute and 9 at five minutes.

## Discussion

Fetoplacental circulation

At the beginning of pregnancy, a series of physiological changes occur at the uterine vascular bed level. The uterine arteries run laterally on both sides of the uterus in a cephalic direction [5,6], branching into the arcuate arteries that irrigate the myometrium during their course. In turn, they divide into radial and straight branches that perfuse the myometrial-endometrial junction, and finally, their most distal branches are the spiral arteries that reach the superficial endometrium [5]. The latter undergo trophoblastic invasion, replacing vascular smooth muscle with epithelial cells and becoming low-resistance systems. In addition, during pregnancy, systemic blood vessels undergo remodeling through angiogenic factors, which leads to a decrease in vascular resistance and an increase in vasodilator factors [3-5]. These vascular changes aim to increase cardiac output and uterine blood flow to ensure adequate fetal perfusion [4].

**Figure 1:**
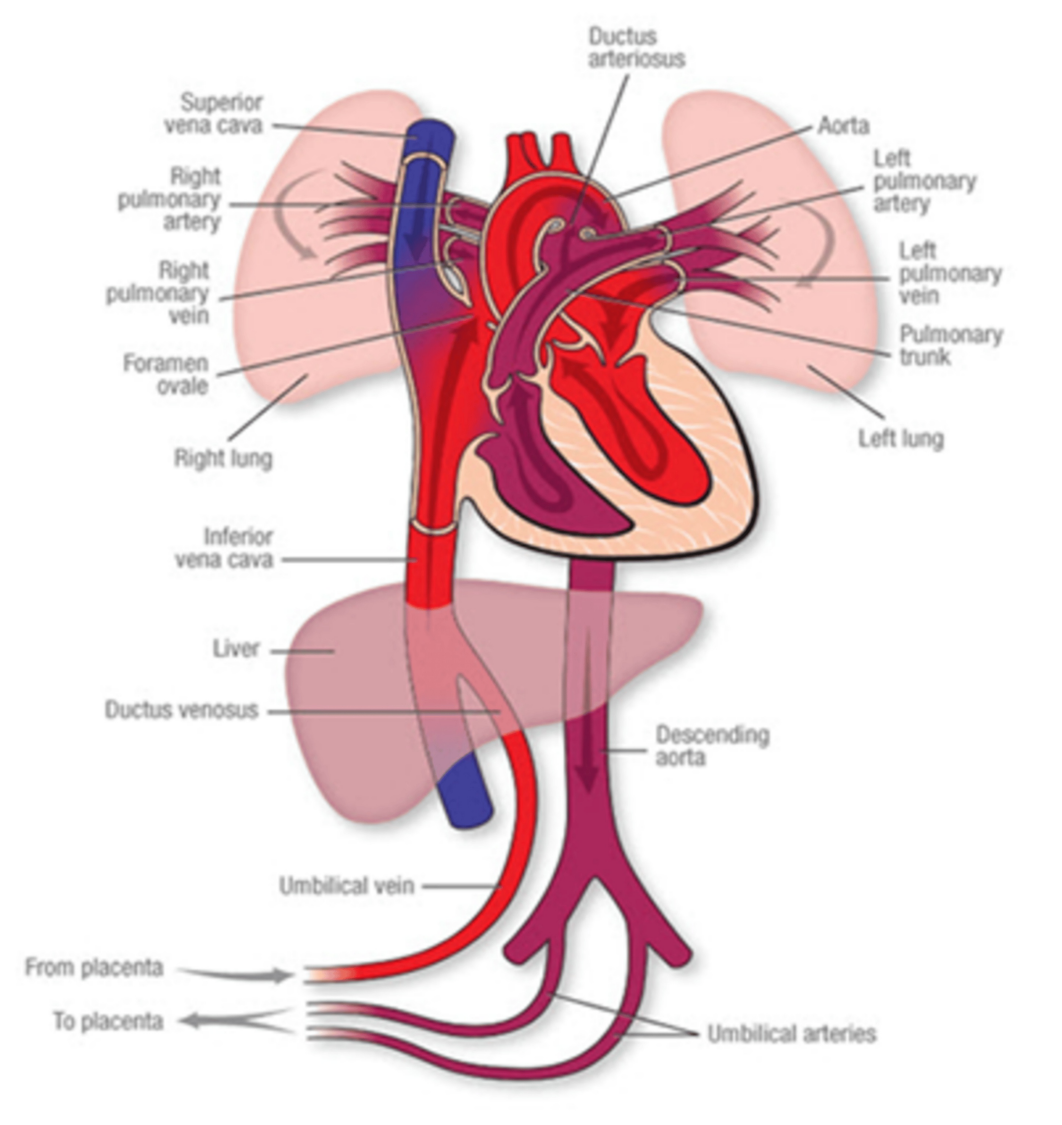
Fetal circulation Reproduced with permission from the American Heart Association [7].

The placenta has two sides, one maternal and one fetal. The maternal side comprises lobes or placentas, which are spiral arteries that dilate until they reach the intervillous space, where the flow is slow to allow adequate maternal-fetal exchange at the level of the villi. The fetal side comprises the umbilical vein and artery, which have low resistance, allowing fetal blood flow to reach the villi without increasing fetal cardiac output [6-8]. Placental fetal circulation is shown in Figure 1. Transplacental oxygen diffusion between uterine and umbilical venous blood does not achieve equilibrium on both sides of the placental barrier. Umbilical venous PaO₂ is less than uterine venous PaO₂ by approximately 14 mmHg. This difference occurs because the villi have a high oxygen consumption and indiscriminately extract oxygen. However, fetal hemoglobin has a high binding affinity for oxygen, which prevents excessive oxygen consumption by the placenta and ensures adequate oxygen content at the fetal level despite low pressure [9].

Mechanism of fetal hypoxia

Fetal hypoxia is a condition characterized by insufficient oxygen delivery to the fetus, which can lead to adverse outcomes, including developmental abnormalities, birth defects, and chronic diseases in adulthood [10]. Oxygen delivery depends on several factors such as gas interchange, uterine flow, placental function, and gas transport at the fetal level. The disruption of any of these mechanisms leads to fetal hypoxia and consequent acidosis [11]. Fetal hypoxia can have either an acute or a chronic course, and the causes are divided into maternal, placental, or fetal. Maternal causes are due to reduced uterine flow or oxygenation [12]. Placental causes include vascular disruption, such as abruptio placentae, or alterations in placental diffusion, such as intrauterine growth restriction. Fetal causes include in-utero cord compression, such as in oligohydramnios, or low fetal oxygen supply, such as in anemia or fetal heart disease.

Mechanical ventilation and fetoplacental circulation

**Figure 2:**
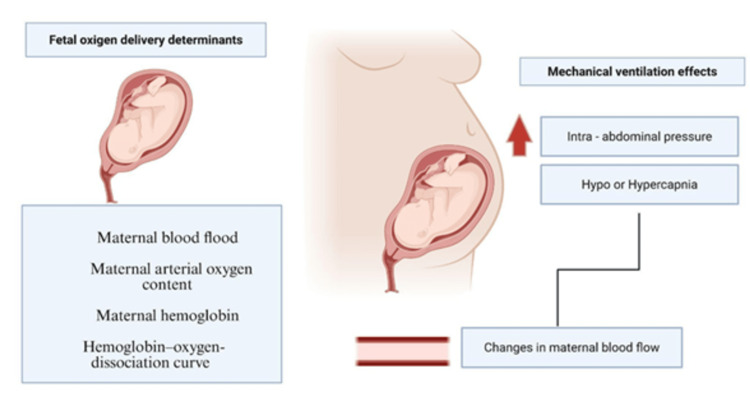
Mechanical ventilation effects and fetal oxygen delivery determinants. Created with BioRender.com [9-15].

Mechanical ventilation in the context of fetal physiology is a complex area involving significant risks and physiological changes. Its effects on fetal circulation are highly variable and contingent upon several factors, including gestational age, the presence of inflammation, and the specific ventilation parameters applied. Fetal oxygen delivery is primarily determined by maternal blood flow, maternal arterial oxygen content, and the characteristics of the hemoglobin-oxygen dissociation curve. Mechanical ventilation, however, can significantly alter the fetal circulation, predominantly by changing the partial pressures of carbon dioxide and oxygen (Figure 2). For instance, a study using fetal sheep demonstrated that an increase in fetal arterial oxygen tension resulting from mechanical ventilation led to a significant increase in the percentage of blood flow shunted to the lungs [13]. While this indicates a clear shift in circulation patterns, the specific and well-defined effects of mechanical ventilation on the human fetal circulation remain poorly understood.

During pregnancy, the respiratory system undergoes many changes. Estrogen and progesterone cause upper airway edema and hyperemia, and uterine enlargement increases intra-abdominal pressure, which alters lung volumes as functional residual capacity decreases and tidal volume increases. Forced expiratory volume in one second (FEV1) and lung compliance remain unchanged, but chest wall compliance decreases [4,5]. Furthermore, the airway in pregnancy is vulnerable to aspiration because of increased intragastric pressure and decreased gastric emptying. Therefore, it is essential to clear and protect the airway as early as possible and perform intubation to ensure good oxygen delivery.

These physiological changes could imply that the pressures of the respiratory system (P peak, plateau, and PEEP) could be tolerated at a higher level than usual. Plateau pressure must be higher, greater than 30 cmH2O, to maintain adequate transpulmonary pressure. In addition, the PEEP level should be higher, usually greater than 10 cmH2O, to avoid collapse and atelectasis. However, it is difficult to determine what pressure limit can be tolerated without inducing lung injury or affecting maternal venous return. This is where mechanical ventilation monitoring systems play an essential role.

One strategy to monitor the pregnant patient's ventilation and avoid ventilator-induced lung injury VILI is the use of the esophageal balloon transpulmonary pressure or intra-abdominal pressure measurement. However, there is no conclusive evidence regarding the best method for pregnant women.

Despite these physiological changes, given the absence of standardized guidelines, ventilatory parameters are configured similarly to those of the general population. Clinical evidence of mechanical ventilation in obstetric patients is scarce and often limited to retrospective studies, with a few prospective studies conducted during the COVID-19 era [14]. In a prospective study conducted in ICUs in Argentina and Colombia, Estenssoro et al. found lung mechanics similar to those of the general population; however, important factors such as PCO_2_, which impact fetoplacental blood flow, were not taken into account [15].

Our ventilatory parameters were similar to those obtained by the surviving patients described by Estenssoro et al., with plateau pressures less than 30 cmH2O and driving pressures less than 14 cmH2O. In our case, plateau pressure ranged from 12 cmH2O to 21 cmH2O and driving pressure ranged from 6 cmH2O to 12 cmH2O. However, fetoplacental Doppler was not obtained; therefore, changes in fetal circulation secondary to mechanical ventilation are unknown.

It is essential to understand the physiological changes of pregnancy in the context of mechanical ventilation in pregnant patients. During this period, increased intra-abdominal pressure and changes in chest mechanics can alter pressure distribution in the respiratory system, suggesting a possible need to tolerate higher-than-usual inspiratory pressures (Pplat and PEEP). However, the absence of well-defined limits that prevent both lung injury and adverse effects on maternal venous return and fetoplacental flow highlights the importance of using more accurate monitoring systems, such as the esophageal balloon or intra-abdominal pressure measurement. Despite these strategies, there is a significant gap in the scientific evidence on their specific use in pregnant women, which limits their clinical applicability.

Furthermore, despite the physiological changes inherent to pregnancy, ventilatory parameters continue to be based on guidelines designed for the general population, largely because of the paucity of specific clinical studies. The available literature is limited, with mostly retrospective studies and few prospective data, such as those conducted during the COVID-19 pandemic, which do not always address crucial variables such as PCO_2_ or fetoplacental circulation. In this regard, there is a clear need for more methodologically rigorous studies exploring the interaction between mechanical ventilation and obstetric physiology.

## Conclusions

In conclusion, the influence of physiological changes during pregnancy on respiratory parameters is well known, which indirectly affects lung mechanics in pregnant women. These changes, although not representing a significant variation in mechanical ventilation parameters according to the limited current evidence, could be indirectly associated with deleterious effects on fetoplacental circulation, given that some ventilation modalities such as PEEP, combined with a context of increased intra-abdominal pressure such as pregnancy itself, could compromise fetal blood flow. Further multicenter studies are needed to clarify the effect of mechanical ventilation on fetoplacental flow.
